# The Association Between Social Media Addiction and Aggressive Behaviors: A Longitudinal and Gender‐Specific Analysis

**DOI:** 10.1002/jad.12454

**Published:** 2024-12-18

**Authors:** S. Lin, M. A. Fabris, C. Longobardi, S. Mastrokoukou

**Affiliations:** ^1^ Department of Psychology Università degli studi di Torino Turin Italy

**Keywords:** aggressive behavior, autoregressive cross‐lagged model, longitudinal, overt aggression, relational aggression, social media addiction

## Abstract

**Introduction:**

Recent evidence demonstrates an association between social media addiction (SMA) and aggressive behaviors; however, the longitudinal relationship between these two variables remains not fully understood. The aim of this study was to examine the longitudinal relationship between SMA and aggressive behaviors (overt and relational aggression) in early adolescence and to identify gender differences in this relationship.

**Methods:**

A sample of 568 Italian early adolescents (52.3% girls; *M*_age_ = 12.59, SD = 0.87) attending middle schools in northern Italy was recruited from different schools. Participants completed an anonymous questionnaire to assess SMA and the perpetration of overt and relational aggressive behaviors. The questionnaire was completed twice with a 1‐year intervals (T1 and T2).

**Results:**

The findings showed that females were at higher risk of SMA and relational aggression, whereas males exhibited higher levels of overt aggression at both time points (T1 and T2). Longitudinal analyses revealed that SMA at T1 was associated with higher likelihoods of both overt and relational aggression at T2 across both genders and that only in females was relational aggression at T1 associated with higher levels of SMA at T2.

**Conclusions:**

The study suggests a relationship between SMA and aggressive behaviors and reveals gender differences in this pattern. Limitations, future directions, and practical implications of the research are discussed.

## 1 Introduction

With the advent of the digital age, social media has become widespread among the population, especially among adolescents, who represent the most frequent users (Marengo et al. 2022). While the impact of social media on adolescents’ psychological adjustment remains controversial (Boniel‐Nissim et al. 2023; Fabris et al. 2024; Popat and Tarrant 2023), many researchers and clinicians warn that excessive or problematic use of social media poses a risk to adolescents’ psychological well‐being (Fabris et al. 2024; Shannon et al. 2022).

In this study, the term “social media addiction” (SMA) refers to excessive or problematic social media use behavior characterized by addiction‐like symptoms and difficulties in self‐regulation, leading to negative consequences for psychological functioning, interpersonal relationships, and other areas of daily life. Specifically, in accordance with Griffiths's model of behavioral addictions (Griffiths 2013), we conceptualize SMA in terms of six salient features: tolerance, withdrawal, preoccupation, neglect of other activities, subjective loss of control, and persistent use despite signs of harm. It is difficult to estimate the prevalence of SMA in adolescents, but some research suggests that the percentage of adolescents suffering from SMA varies between 2% and 14%, with the Italian population appearing to be more affected than other European populations (Boer et al. 2020; Boniel‐Nissim et al. 2023).

Adolescents seem to be at particularly high risk of developing SMA. This increased risk can be understood by considering the particular role that social media plays in early adolescence and its ability to respond to the psychological and social needs typical of this developmental stage. Indeed, adolescents use social media for fun and to pass time, but it also provides them with a unique opportunity to explore their identity and to reveal and express different characteristics of themselves (Michikyan and Suárez‐Orozco 2016). Although the relationship with parents still plays an important role, in early adolescence the peer group gradually becomes the preferred source of emotional and social support and the place where intimate relationships develop (Badenes‐Ribera et al. 2019). In this sense, social media allow adolescents to constantly stay in touch with their peers, seek and maintain new friendships and satisfy their need to belong to the peer group (Fabris et al. 2024; Hu et al. 2023).

Moreover, social media serve as an important tool for daily interaction, especially for early adolescents, (Antheunis, Schouten, and Krahmer 2016), particularly in relation to the classroom group (Marengo et al. 2021). Finally, early adolescents may be more reliant on Internet use (including social media) compared to later adolescents, as they tend to be less mature and, therefore, less able to cope with challenges and stressors in the real world. Consequently, they are more likely to escape to the online world (Hu et al. 2023), an environment that is not devoid of challenges and stressful or dangerous situations, as documented in the literature (Longobardi et al. 2021).

However, the literature on SMA has primarily focused on late adolescence, and little research has been conducted on the specific period of early adolescence (Lin et al. 2024), especially when examining the possible influence of problematic social media use on the development of problem behaviors, such as aggression.

### 1.1 Aggressive Behavior in Early Adolescence

Aggressive behaviors during adolescence are considered a significant social concern, particularly within educational and school settings (Longobardi, Prino, et al. 2019; Longobardi, Badenes‐Ribera, et al. 2019; Xing et al. 2023). Aggression refers to any behavior intentionally aimed at causing harm to others (Anderson and Bushman 2002; Crick 1996). Literature classifies aggressive behaviors into two primary forms: overt aggression and relational aggression (Little et al. 2003). While both forms share similar underlying motivations, they differ in the “vehicles of harm” they employ (Crick, Ostrov, and Kawabata 2007).

Overt aggression is characterized by externalized behaviors intended to harm others through physical actions—such as hitting, pushing, or kicking—or through verbal attacks (e.g., insults and threats). In contrast, relational aggression is a pattern of behaviors aimed at harming the victim by damaging relationships or social status through indirect means, such as spreading rumors, social exclusion, or ostracism from peers. Unlike overt aggression, which typically involves direct physical and/or verbal interaction between the aggressor and the victim, relational aggression exploits social connections or friendship statuses as tools to inflict harm (Crick, Ostrov, and Kawabata 2007).

Peer group dynamics can contribute to the reinforcement of overt aggression, particularly in male‐dominated contexts where physical displays of dominance are not only socially accepted but also rewarded (Pellegrini 2002). In such an environment, aggressive behavior may become normalized, functioning as a strategy to attain or maintain social status within the group (Kim and Cillessen 2023). Moreover, research suggests that relational aggression is positively associated with adolescents’ perceived popularity and social preference, whereas overt aggression tends to be negatively correlated with these outcomes (Prinstein and Cillessen 2003). Furthermore, evidence suggests that overt aggression is associated with higher levels of emotional dysregulation, whereas relational aggression is not (Marsee and Frick 2007). Boys tend to engage in more direct forms of aggression, while relational aggression is more frequently associated with females, as some studies suggest (Card et al. 2008)

Although there are some differences, both forms of aggression have been consistently linked to negative developmental, psychosocial, and academic outcomes in adolescents (Crick 1996; Prinstein, Boergers, and Vernberg 2001). Therefore, it is crucial to identify the factors that contribute to the emergence and persistence of aggressive behaviors. A deeper understanding of these dynamics may facilitate the development of more targeted interventions and support systems, which can mitigate these behaviors and promote healthier social interactions among adolescents.

### 1.2 SMA and Aggressive Behaviors

Recent evidence suggests a link between SMA and aggressive behaviors in adolescents (Hussain et al. 2023; Lin et al. 2024; Kırcaburun et al. 2019; Rustamov et al. 2023; Wong et al. 2022) and young adults (Bersani et al. 2022; Kırcaburun et al. 2019). These studies highlight SMA as a risk factor for heightened aggressive behavior. Generally, social media use is considered a contributor for youth violence (Patton et al. 2014), and frequent exposure to violent online content may elevate the risk of engaging in violent behavior (Ko et al. 2009; Vannucci et al. 2020). This interpretation is supported by the following evidence.

First, from a neuropsychological perspective, both problematic social media use and aggressive behaviors share common biological underpinnings, including neural structures such as the prefrontal cortex and limbic system, as well as neurotransmitters like dopamine, noradrenaline, serotonin, opioids, and nicotine (Bersani et al. 2022). These brain areas play critical roles in executive functions and impulse inhibition (prefrontal cortex) and emotional regulation processes (limbic system). This overlap underscored a potential strong relationship between these two behavioral dimensions.

Second, according to frustration‐aggression theory (Berkowitz 1989), individuals who feel frustrated are more likely to engage in maladaptive behaviors, particularly aggressive behaviors. This theory posits that frustration is associated with negative emotional tension, and that aggression can be an effective way to rapidly reduce the negative effects of frustration, especially when individuals lack more functional strategies for regulating their negative affective states. In such cases, frustration‐induced emotional distress may intensify the likelihood of aggressive responses (Kruglanski et al. 2023). The online context, especially with frequent use, often provides situations that may heighten feelings of frustration and irritability (Fabris et al. 2020; Lin et al. 2024). For instance, problematic social media use in adolescence has been associated with an elevated risk of online victimization (Longobardi et al. 2020, 2021), which in turn may increase negative feelings and hostility, thereby exacerbating frustration (Wang, Li, and Xia 2023). Moreover, adolescents who extensively use social media may develop negative feelings such as envy, triggered by persistent social comparisons with individuals perceived as idealized models of perfection (Radovic et al. 2017).

Third, adolescents with SMA tend to isolate themselves from real‐world interactions, which may expose them to criticism and reproach from adults (parents and teachers) and peers, thereby reinforcing a sense of hostility and negative feelings that can contribute to aggressive behavior (Lin et al. 2024). Along these lines, some evidence suggests that adolescents with internet‐related addiction have a lower frustration tolerance (Ko et al. 2014), which makes them more likely to respond to negative feelings and stress with aggressive behaviors.

Finally, research shows that individuals with high levels of SMA often exhibit poorer social skills and deficits in social competencies (Dredge and Schreurs 2020), which indicates that individuals with high levels of SMA may be at greater risk of resorting to aggressive behaviors to resolve conflicts in interpersonal relationships (Rustamov et al. 2023). Moreover, longitudinal studies (Fitzpatrick & Boers 2022) suggest that prolonged social media use can have a negative impact on the development of empathic and prosocial behaviors, presumably due to the decrease in face‐to‐face interactions.

However, the cross‐sectional nature of the studies presented here limits our ability to understand the temporal sequencing relationships between the two variables, and although the majority of available studies seem to point to SMA as a predictor of aggressive behavior, alternative hypotheses could also be considered. For example, following the Uses and Gratifications Theory (Katz, Blumler, and Gurevitch 1973), Wong and colleagues (2022) hypothesized that adolescents with high levels of appetitive aggression (i.e., high motivation to engage in aggressive behavior for rewards such as more power or higher social status in the peer group) would exhibit higher problematic social media use. In this sense, social media could become a context in which adolescents satisfy their needs through aggressive behavior, and this would reinforce social media use and contribute to the development of addictive behavior. On the other hand, retreating to the online context could become a strategy for aggressive adolescents to compensate for their negative feelings and they might consider social media as a context in which they can behave as they wish without facing any consequences (Hussain et al. 2023).

In summary, despite a growing number of studies demonstrating an association between SMA and aggressive behaviors, further research is needed to understand the temporal sequencing relationship between the two constructs, especially in early adolescence, where the association may be stronger (Ko et al. 2014). In addition, studies on this topic have mainly focused on the relationship between SMA and aggressive behaviors in the online environment, while there is little evidence on the relationship between SMA and aggressive behavior in the real world (Lin et al. 2024) and no study have examined the relationship between SMA and the two dimensions of aggressive behavior: overt and relational aggression.

### 1.3 Gender Differences

The literature consistently indicates that females are at higher risk of exhibiting SMA (Su et al. 2020; Xu et al. 2022). Typically, females are more interpersonally oriented and report greater sensitivity to social cues and evaluation of interpersonal relationships (Proverbio, Zani, and Adorni 2008; Su et al. 2020). This tendency is also reflected in the way females use social media compared to males. In fact, there is evidence that females use social media more often as a tool for social interaction, while males use it predominantly for entertainment purposes (Chae, Kim, and Kim 2018). In addition, females are more likely to resort to social media to fill emotional voids when they are depressed and their social needs are not fully met, which puts them at high risk of developing SMA (Su et al. 2020).

In terms of aggressive behavior, the data are less consistent and, in general, the literature shows that males tend to report higher levels of overt aggression, while females tend to report higher or equal levels of relational aggression compared to males (Card et al. 2008; O'Dell, Charles, and Barry 2024). Given these differences, it is important to examine the association between aggressive behaviors and SMA in males and females to determine similarities and differences in this regard.

### 1.4 The Aim of the Study

The aim of the study is to examine the longitudinal relationships between SMA and forms of aggression (overt and relational) among early adolescents, taking into account gender differences. First, we hypothesize that female adolescents will report higher levels of SMA and show more relational aggression, whereas we expect male adolescents to show more overt aggression. Furthermore, we hypothesize that there is a bidirectional relationship between SMA and both forms of aggression (i.e., relational and overt).

## 2 Method

### 2.1 Procedures and Participants

After receiving ethical approval from the ethics committee at the authors’ affiliated university, research assistant began contacting middle schools located in northern Italy for data collection. These schools were contacted through the researchers’ personal networks or publicly available contact information (e.g., e‐mail). Before data collection, consent forms were signed by all the participants and their parents. Participants were students from middle schools. In the first wave of the data collection,642 students (331 girls, 51.6%) participated in this study. After a 1‐year interval, 575 students (299 girls, 52.0%) were followed up in the second wave of data collection. Participants with incorrect identification information were excluded. The final sample consisted of 568 students (297 girls, 52.3%) with a mean age of 12.59 years (SD = 0.87) in the second wave.

### 2.2 Measures

#### 2.2.1 SMA

The SMA in both Time 1 and Time 2 was measured with the Bergen Social Media Addiction Scale (BSMAS) (Andreassen et al. 2016; Monacis et al. 2017). The BSMAS is a 5‐point scale (1 = *Very rare*, 5 = *Very often*). Students were required to rate six items (e.g., “How often have you become restless or troubled if you have been prohibited from using social media?”) that were developed based on the six components of addiction (i.e., salience, mood change, tolerance, withdrawal, conflict, and relapse). The final score of the SMA level was calculated as the sum of the ratings on all six items, with higher values representing higher levels of SMA. In the original and Italian sample, the internal consistency coefficients of BSMAS were both 0.88 (Andreassen et al. 2016; Monacis et al. 2017). In the current sample, the internal consistency coefficient of BSMAS was acceptable at both Time 1 (Cronbach's *α* = .75) and Time 2 (Cronbach's *α* = .72).

#### 2.2.2 Aggressive Behaviors

A self‐report scale by Little et al. (2003) was adopted to measure aggressive behaviors. This scale consists of 36 items, measuring both overt aggressive behaviors (18 items, e.g., “I'm the kind of person who hits, kicks, or punches others.”) and relational aggressive behaviors (18 items, e.g., “If others upset or hurt me, I often tell my friends to stop liking them.”). Students were required to rate these items on a 4‐point scale (1 = *Not at all true*, 4 = *Completely true*). The final scores for overt aggression and relational aggression were computed by summing the ratings of all items within each subscale, with higher scores indicating more aggressive behaviors. In the original sample, the internal consistency coefficients of the subscales ranged from 0.62 to 0.84 (Little et al. 2003). In the current sample, the internal consistency coefficients of the two subscales were satisfactory at both Time 1 (overt aggression: Cronbach's *α* = .86; relational aggression: Cronbach's *α* = .86) and Time 2 (overt aggression: Cronbach's *α* = .86; relational aggression: Cronbach's *α* = .87).

#### 2.2.3 Data Analysis

SPSS version 29.0 and Mplus version 8.3 (Muthén and Muthén 1998–2017) were used to analyze the data. First, descriptive statistics (means and standard deviations) and correlational statistics were calculated to preliminarily explore the relationships between the variables of interest. Then, to explore the longitudinal relationships and temporal order between SMA and aggressive behaviors across two time points, an autoregressive cross‐lagged panel model (Selig and Little 2012) was performed on the two waves of the data in Mplus. This model explores both the autoregressive effects and cross‐lagged effects between variables across time. The autoregressive effect represents the stability of the same variable over time, and the cross‐lagged effect indicates the directionality of the variables over time. Lastly, a multi‐group analysis was conducted to explore the potential differences in the final model across genders. Initially, all the paths were constrained to be equal for girls and boys. Subsequently, these path constraints were released one at a time to assess whether the model's goodness‐of‐fit significantly improved by releasing each constraint. To assess the goodness of model fit, the following fit indices and their respective acceptable cut‐off points were utilized: (1) *χ* ^2^ statistics and degrees of freedom; (2) Root Mean Square Error of Approximation (RMSEA < 0.08, McDonald and Ho 2002); (3) Comparative Fit Index (CFI > 0.95, Hu and Bentler 1999); (4) Tucker–Lewis Index (TLI > 0.95, Hu and Bentler 1999); and (5) Standardized Root Mean Square Residual (SRMR < 0.08, Hu and Bentler 1999).

## 3 Results

### 3.1 Descriptive Statistics and Correlations

The descriptive statistics (means and standard deviations) and correlations between the studied variables are shown in Table 1. Overall, all the main studied variables in the model were consistently correlated with each other across time (*r* ranged from .53 to .63, all *p* < .001). SMA was positively correlated with overt and relational aggression at both Time 1 and Time 2. In addition, at both Time 1 and Time 2 girls tended to have higher levels of SMA (*r*_T1_ = –.15, *p* < .001; *r*_T2_ = –.19, *p* < .001) and relational aggression (*r*_T1_ = –.09, *p* < .05; *r*_T2_ = –.09, *p* < .05), while boys tended to have higher levels of overt aggression (*r*_T1_ = .19, *p* < .001; *r*_T2_ = .18, *p* < .001) at both Time 1 and Time 2.

**Table 1:** Descriptive statistics and correlations of the variables.

Variables	1	2	3	4	5	6	7
1. Gender	—						
2. SMA_T1	–0.15***	—					
3. SMA_T2	–0.19***	0.63***	—				
4. Overt aggression_T1	0.19***	0.28***	0.22***	—			
5. Overt aggression_T2	0.18***	0.28***	0.31***	0.61***	—		
6. Relational aggression_T1	–0.09*	0.42***	0.36***	0.64***	0.41***	—	
7. Relational aggression_T2	–0.09*	0.36***	0.39***	0.40***	0.63***	0.53***	—
*M*	0.48	12.42	12.66	25.47	25.85	25.65	25.64
SD	0.50	4.97	4.74	6.52	6.60	6.88	6.81

*Note:* Gender was coded as 0 = *girls*, 1 = *boys*.

Abbreviations: SMA, social media addiction; T1, Time 1; T2, Time 2.

*** *p* < .001.

### 3.2 Longitudinal Relationship Between SMA and Aggression

**Figure 1:**
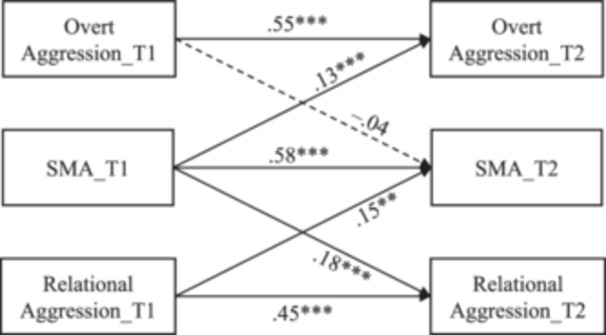
The autoregressive cross‐lagged model between social media addiction and two forms of aggressive behaviors (overt aggression and relational aggression) in full sample. *Note: N* = 568; the path coefficients were standardized; the dotted lines indicate nonsignificant paths; the within‐time correlations were added but not shown to keep the figure concise.

The autoregressive cross‐lagged panel model was employed to explore the longitudinal relationship between SMA and aggressive behaviors. The goodness of fit for the model (shown in Figure 1) was acceptable: *χ* ^2^/*df* = 3.07, RMSEA = 0.06, CFI = 0.996, TLI = 0.975, and SRMR = 0.02. All the autoregressive paths were significant (*β* ranged from .45 to .58, *p* < .001), which indicated the stability of the studied variables across two time points. For the cross‐lagged pathways between SMA and the two forms of aggression, the results indicated that SMA at Time 1 positively predicted both overt aggression (*β* = .13, *p* < .001) and relational aggression (*β* = .18, *p* < .001) at Time 2. In addition, relational aggression at Time 1 positively predicted SMA at Time 2 (*β* = .15, *p* < .001), whereas overt aggression at Time 1 did not predict SMA at Time 2 (*β* = –0.04, *p* = .33). Therefore, a bidirectional relationship between SMA and relational aggression, as well as a unidirectional relationship between SMA and overt aggression, were discovered.

### 3.3 Gender Differences

**Figure 2:**
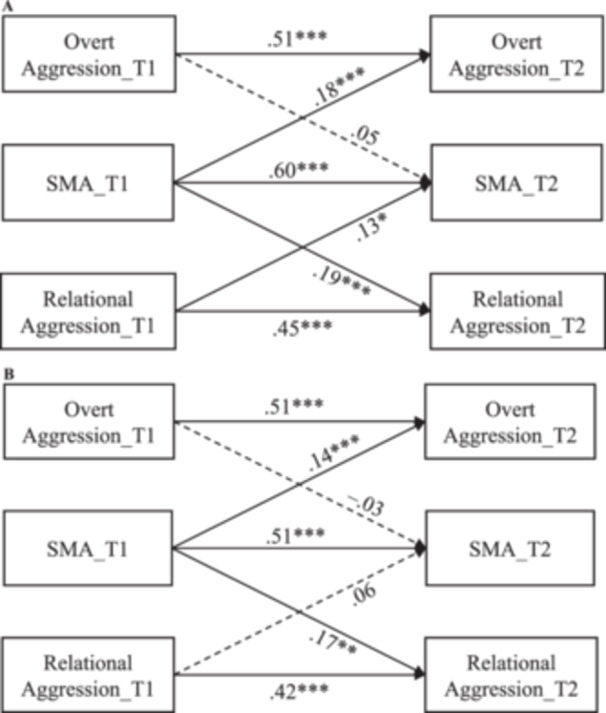
(A) The autoregressive cross‐lagged model between social media addiction and two forms of aggressive behaviors (overt aggression and relational aggression) in GIRLS. *Note: N*_girls_ = 297; the path coefficients were standardized; the dotted lines indicate nonsignificant paths; the within‐time correlations were added but not shown to keep the figure concise. (B) The autoregressive cross‐lagged model between social media addiction and two forms of aggressive behaviors (overt aggression and relational aggression) in BOYS. *Note: N*_boys_ = 271; the path coefficients were standardized; the dotted lines indicate nonsignificant paths; the within‐time correlations were added but not shown to keep the figure concise.

To explore gender differences, a multi‐group analysis was conducted. All the paths in the autoregressive cross‐lagged model were constrained to be equal across girls and boys, and the model fit indices were as follows: *χ* ^2^/*df* = 2.02, RMSEA = 0.06, CFI = 0.982, TLI = 0.975, and SRMR = 0.09. Then, the constraints on the paths within the autoregressive cross‐lagged model were individually released to ascertain whether such releases would result in a significant improvement in model fit. It was found that the releases of constraints on three paths yielded a better model fit, including the path from Overt Aggression_T1 to SMA_T2 (∆*χ* ^2^(1) = 7.82, *p* < .01), the path from SMA_T1 to SMA_T2 (∆*χ* ^2^(1) = 9.53, *p* < .01), and the path from Relational Aggression_T1 to SMA_T2 (∆*χ* ^2^(1) = 8.69, *p* < .01). Subsequently, these paths were freed in the final gender‐moderated model, which fitted well with the data (*χ* ^2^/*df* = 1.46, RMSEA = 0.04, CFI = 0.993, TLI = 0.989, and SRMR = 0.06). As illustrated in Figure 2A (girls’ model) and Figure 2B (boys’ model), girls’ SMA over two time points was more stable, and the bidirectionality between SMA and relational aggression was found only among girls.

## 4 Discussion

The aim of this study was to investigate the longitudinal association between SMA and two forms of aggressive behavior (i.e., overt aggression and relational aggression) in a sample of early adolescents. Our data suggest that females have a higher risk of reporting SMA. This finding is consistent with previous literature highlighting a greater predisposition of females to problematic social media use (Su et al. 2020), which is likely due to their higher sensitivity to interpersonal relationships and social stimuli (Su et al. 2020). In addition, compared to boys, girls tend to use social media more often as a response to feelings of emptiness when their social needs are not fully met in real life, which puts them at higher risk for SMA (Chae, Kim, and Kim 2018).

At both time points (T1 and T2), it emerges that females tend to have higher relational aggression, while males tend to have higher overt aggression. These data are consistent with previous literature (Card et al. 2008), although it should be noted that the data regarding the association between male gender and overt aggression are consistent, whereas the data demonstrating a stronger association between relational aggression and female gender are mixed (Card et al. 2008; Smith, Rose, and Schwartz‐Mette 2010). It is likely that gender differences in relational aggression become more pronounced as individuals grow and enter adolescence (Archer 2004; Smith, Rose, and Schwartz‐Mette 2010). Undoubtedly, females are socialized away from overt aggression more strongly than boys (Archer 2004). In addition, girls’ verbal and social‐perspective taking skills develop faster than boys’ (Crick et al. 1998), which allows females to resort to indirect forms of aggression, such as relational aggression, earlier than males (Smith, Rose, and Schwartz‐Mette 2010).

With regard to the two forms of aggressive behavior, the analyses indicate that overt and relational aggression at T1 tend to be associated with overt or relational aggression at T2, although this association appears moderate. The moderate correlation between T1 and T2 suggests that while these aggressive behaviors demonstrate some level of stability over time, external factors may contribute to changes in their intensity or expression. Situational factors, such as shifts in peer group dynamics, developmental changes, or the increasing centrality of social media in adolescents’ lives, could play a role in this moderate association. As adolescents progress through this developmental period, they may experience changes in their social environment, which could either reinforce or mitigate these aggressive tendencies. Thus, peer influence, social media use, and school‐related experiences might amplify or dampen these behaviors, leading to the moderate rather than strong correlation observed between T1 and T2. The time span considered in this longitudinal study limits our interpretation of this result. However, it may be that the forms of aggression studied are quite stable in early adolescence and that there may be situational or dispositional factors that increase aggression over time, which could be incorporated into the theoretical model in future studies.

The analyses conducted on the full sample indicate that SMA is longitudinally associated with aggressive behaviors, both relational and overt forms. These data are consistent with previous literature that has found a relationship between SMA and aggressive behaviors in the real world (Lin et al. 2024). However, research on the specific relationship between SMA and aggressive behaviors in early adolescents is still limited, and the available studies have used cross‐sectional approaches and general measures of aggressive behavior (Lin et al. 2024). Our study examined two different forms of aggressive behavior and found that SMA is associated with both forms of aggressive behavior 1 year later, adding new evidence to the current literature.

Although possible mediators were not considered in our study, there are several reasons that could explain this association. It is possible that adolescents with SMA have poor social skills (Savcı and Aysan 2018) and a lower capacity for empathy (Dalvi‐Esfahani et al. 2021; Fitzpatrick and Boers 2022), which could lead adolescents with high SMA scores to resort more frequently to forms of aggression to manage interpersonal conflict. In addition, adolescents with SMA may experience more negative feelings and greater frustration due to increased exposure to online victimization experiences (Longobardi et al. 2020, 2021) and constant social comparisons (Radovic et al. 2017). In this emotional context, it is possible that adolescents with SMA are at higher risk of experiencing frustration, which increases their resort to aggressive behaviors in the offline environment. Our research, therefore, indicates a longitudinal relationship between SMA and aggressive behaviors. However, we only found a bidirectional relationship with SMA for relational aggression. Indeed, SMA predicts not only relational aggression at T2, but also vice versa. It is possible that aggressive adolescents use social media more frequently to satisfy their needs, such as the desire for popularity or greater power within peer groups (Wong et al. 2022). However, it was found that only relational aggression, but not overt aggression at T1, predicted SMA at T2. This finding is intriguing and we believe further research is needed to understand these.

It has been widely demonstrated in the literature that aggressive children and adolescents tend to be rejected by their peers (LoParo et al. 2023; Marengo et al. 2021). And recent evidence suggests that peer rejection is more strongly associated with relational aggression, especially in early adolescence (Yue and Zhang 2023). Adolescents who are rejected by peers are more likely to feel lonely, isolated, and excluded (Cheek, Reiter‐Lavery, and Goldston 2020; Morese et al. 2024). Adolescents who feel rejected and socially excluded tend to experience painful feelings (Morese et al.^2024^) and anger (Yue and Zhang 2023) and may compulsively turn to social media to relieve their emotional pain and compensate for their frustrated needs for relationships and belonging (Fabris et al. 2020; Yue and Zhang 2023). In this direction, for example, there is evidence that anger is a mediating factor between social exclusion and SMA in adolescents (Yue and Zhang 2023). Therefore, it is possible that adolescents who exhibit relational aggression in early adolescence are more likely to be excluded or isolated from peer groups, leading them to resort more to social media to compensate for their feelings of loneliness and distress, which in turn puts them at higher risk for SMA. It should also be noted that relational aggression, compared to overt aggression, involves manipulating others (e.g., spreading rumors, excluding someone), so the nature of relational aggression emanates from and is amplified by social networks (Low, Polanin, and Espelage 2013). Moreover, in early adolescence, difficulties that occur in the real world in peer relationships can easily reverberate online (such as class groups in WhatsApp), where forms of relational aggression are the most prevalent forms of aggression (Marengo et al. 2021). In this sense, relational aggression may find a way to express and perpetuate itself in social media, and this may help to explain a significant link between relational aggression and SMA.

Finally, it should be noted that the reciprocal relationship between relational aggression and SMA was only observed in the female sample and not in the male sample. Thus, unlike in the female adolescent group, relational aggression is not a long‐term predictor of SMA in the male adolescents. This result needs further investigation, but the explanation may reflect the gender differences mentioned above. One explanation could be that female adolescents are more prone to relational aggression during adolescence than males and that this aggression finds a way to express or reinforce itself through social media, leading to compulsive behavior, likely as a result of frustrated relational feelings or the need to achieve social goals such as higher social status through aggressive behavior. It is, therefore, possible that boys report more overt aggression and express it more frequently in real life, reducing the tendency to develop SMA.

In conclusion, our study adds new findings to the current literature in understanding possible factors in the development of SMA and, in particular, explores the relationship between aggressive behavior and SMA. The study we propose is longitudinal, whereas the data we have on the relationship between SMA and aggressive behavior mainly come from cross‐sectional studies. Moreover, our study suggests that it is important to consider different forms of aggressive behavior and the role of gender in the relationship between aggressive behavior and SMA. Last but not least, our data attempt to expand our knowledge of early adolescence, a developmental period that has been neglected in the study of the relationship between aggressive behavior and SMA. However, this is a limitation considering that early adolescence is a critical developmental period characterized by major physical, psychological, and social changes. A developmental period in which aggression reaches its peak and in which adolescents are particularly vulnerable to the risk of developing forms of behavioral addiction. During this transitional period between childhood and adolescence, individuals explore their identity and develop greater autonomy from the family, while the peer group becomes the main source of social support. During this time, smartphones with internet access become particularly popular with boys and girls. They seem to use social media to maintain their relationships and satisfy their relationship needs. Overall, our study contributes to the literature by identifying a relationship between SMA and aggressive behaviors and finding significant gender differences.

Our data support several potential directions for intervention and prevention in relation to aggressive behaviors in adolescents. In particular, it is important to continue research on the relationship between problematic social media use and aggressive behaviors in early adolescence and to identify the possible mechanisms. In this way, it will be increasingly possible to develop programs to raise awareness among adolescents, families, and educators about the potential impact of SMA on social behavior. Prevention and intervention measures with adolescents should also take into account the gender differences identified here to develop gender‐specific strategies. While it is important to talk to adolescents about the negative effects of forms of aggression on the psychological adjustment of both victims and aggressors, effective prevention should consider the socialization processes that seem to link the male gender to a greater manifestation of overt aggression and the female gender to a greater manifestation of relational aggression. Girls, in particular, seem to be more sensitive to interpersonal conflicts and issues of social exclusion. Therefore, it might be useful to focus the discussion on how problematic social media use might influence their social behavior by putting them at greater risk of rejection and exclusion through the expression of relationally aggressive behavior. In addition, supporting girls to develop more positive and empathetic relationships with peers could improve interpersonal relationships and reduce feelings of exclusion, potentially helping to reduce the risk of problematic social media use. In parallel, boys could benefit from interventions that focus on anger management, emotion regulation, and improving empathy skills to reduce aggressive overt behavior and prevent the onset of problems such as SMA. In addition, mental health and educational professionals working with adolescents can assess the presence of aggressive behaviors and overall interpersonal functioning in adolescents exhibiting symptoms of SMA. Conversely, professionals can examine social media use in adolescents who exhibit aggressive behaviors, particularly the presence of excessive and problematic social media use. This could be very useful for school psychologists, for example, who are entrusted with the assessment and psychological support of young people who commit aggressive acts in a school context.

### 4.1 Limits and Future Direction

Despite the contribution of our study to the understanding of the relationship between SMA and aggressive behavior in early adolescence, our study must be considered in light of its methodological limitations. First, the large sample of adolescents we recruited is a convenience sample that is not representative of the Italian adolescent population. Therefore, future studies could consider representative samples and also try to investigate possible differences or similarities with other cultural contexts. Second, this study focuses on early adolescence. Future studies could propose comparisons with other developmental periods, such as late adolescence and early adulthood to identify specifics linked to different developmental stages. The sample we recruited is a general sample, while future studies could examine adolescents diagnosed with SMA. Third, another limitation relates to the instruments used. Namely, we only used self‐report instruments, which could favor bias in terms of text comprehension, memory, or social desirability. Future research might adopt third‐party observers or other research instruments. Fourth, the time interval considered could be extended to assess the relationship between the variables over a longer period of time with more timepoints. Finally, although our primary aim was to help clarify the longitudinal relationship between SMA and aggressive behaviors, no mediating or moderating factors were examined in our study. Therefore, subsequent replications of the study could enrich the research protocol with additional variables that might explain the mechanisms by which SMA and aggressive behavior influence each other, or identify overlooked factors that might influence both variables.

## Ethics Statement

All procedures performed in studies involving human participants were in accordance with the ethical standards of the institutional and/or national research committee and with the 1964 Helsinki declaration and its later amendments or comparable ethical standards.

## Consent

Informed consent was obtained from all individual participants included in the study.

## Conflicts of Interest

The authors declare no conflicts of interest.

## Data Availability

For this research, we used SPSS 29 software available from our University. The data that support the findings of this study are available from the corresponding author upon reasonable request.

## References

[jad12454-bib-0001] Anderson, C. A. , and B. J. Bushman . 2002. “Human Aggression.” Annual Review of Psychology 53, no. 1: 27–51. 10.1146/annurev.psych.53.100901.135231 11752478

[jad12454-bib-0002] Andreassen, C. S. , J. Billieux , M. D. Griffiths , et al. 2016. “The Relationship Between Addictive Use of Social Media and Video Games and Symptoms of Psychiatric Disorders: A Large‐Scale Cross‐Sectional Study.” Psychology of Addictive Behaviors 30, no. 2: 252–262. 10.1037/adb0000160. 26999354

[jad12454-bib-0003] Antheunis, M. L. , A. P. Schouten , and E. Krahmer . 2016. “The Role of Social Networking Sites in Early Adolescents’ Social Lives.” Journal of Early Adolescence 36, no. 3: 348–371. 10.1177/0272431614564060.

[jad12454-bib-0004] Archer, J. 2004. “Sex Differences in Aggression in Real‐World Settings: A Meta‐Analytic Review.” Review of General Psychology 8, no. 4: 291–322. 10.1037/1089-2680.8.4.291.

[jad12454-bib-0005] Badenes‐Ribera, L. , M. A. Fabris , F. G. M. Gastaldi , L. E. Prino , and C. Longobardi . 2019. “Parent and Peer Attachment as Predictors of Facebook Addiction Symptoms in Different Developmental Stages (Early Adolescents and Adolescents).” Addictive Behaviors 95: 226–232. 10.1016/j.addbeh.2019.05.009. 31103243

[jad12454-bib-0006] Berkowitz, L. 1989. “Frustration‐Aggression Hypothesis: Examination and Reformulation.” Psychological Bulletin 106, no. 1: 59–73. 2667009 10.1037/0033-2909.106.1.59

[jad12454-bib-0007] Bersani, F. S. , B. Barchielli , S. Ferracuti , et al. 2022. “The Association of Problematic Use of Social Media and Online Videogames With Aggression is Mediated By Insomnia Severity: A Cross‐Sectional Study in a Sample of 18‐to 24‐Year‐Old Individuals.” Aggressive Behavior 48, no. 3: 348–355. 10.1002/ab.22008. 34870339

[jad12454-bib-0008] Boer, M. , R. J. J. M. Van Den Eijnden , M. Boniel‐Nissim , et al. 2020. “Adolescents’ Intense and Problematic Social Media Use and Their Well‐Being in 29 Countries.” Journal of Adolescent Health 66, no. 6: S89–S99. 10.1016/j.jadohealth.2020.02.014. PMC7427320 32446614

[jad12454-bib-0009] Boniel‐Nissim, M. , J. Tynjälä , I. Gobiņa , et al. 2023. “Adolescent Use of Social Media and Associations With Sleep Patterns Across 18 European and North American Countries.” Sleep Health 9, no. 3: 314–321. 10.1016/j.sleh.2023.01.005. 36804326

[jad12454-bib-0010] Card, N. A. , B. D. Stucky , G. M. Sawalani , and T. D. Little . 2008. “Direct and Indirect Aggression During Childhood and Adolescence: A Meta‐Analytic Review of Gender Differences, Intercorrelations, and Relations to Maladjustment.” Child Development 79, no. 5: 1185–1229. 10.1111/j.1467-8624.2008.01184.x. 18826521

[jad12454-bib-0011] Chae, D. , H. Kim , and Y. A. Kim . 2018. “Sex Differences in the Factors Influencing Korean College Students’ Addictive Tendency Toward Social Networking Sites.” International Journal of Mental Health and Addiction 16: 339–350. 10.1007/s11469-017-9778-3.

[jad12454-bib-0012] Cheek, S. M. , T. Reiter‐Lavery , and D. B. Goldston . 2020. “Social Rejection, Popularity, Peer Victimization, and Self‐Injurious Thoughts and Behaviors Among Adolescents: A Systematic Review and Meta‐Analysis.” Clinical Psychology Review 82: 101936. 10.1016/j.cpr.2020.101936. 33128964

[jad12454-bib-0013] Crick, N. R. 1996. “The Role of Overt Aggression, Relational Aggression, and Prosocial Behavior in the Prediction of Children's Future Social Adjustment.” Child Development 67, no. 5: 2317–2327. 10.1111/j.1467-8624.1996.tb01859.x. 9022243

[jad12454-bib-0014] Crick, N. R. , J. M. Ostrov , and Y. Kawabata . 2007. “Relational Aggression and Gender: An Overview.” In The Cambridge Handbook of Violent Behavior and Aggression, edited by. D. J. Flannery , A. T. Vazsonyi , I. D. Waldman , D. J. Flannery , A. T. Vazsonyi , and I. D. Waldman , 245–259. Cambridge, UK: Cambridge University Press.

[jad12454-bib-0015] Crick, N. R. , N. E. Werner , J. F. Casas , et al. 1998. “Childhood Aggression and Gender: A New Look at an Old Problem.” In Nebraska Symposium on Motivation, edited by D. Bernstein , 75–141. Lincoln, NE: University of Nebraska Press. 10752059

[jad12454-bib-0016] Dalvi‐Esfahani, M. , A. Niknafs , Z. Alaedini , H. Barati Ahmadabadi , D. J. Kuss , and T. Ramayah . 2021. “Social Media Addiction and Empathy: Moderating Impact of Personality Traits Among High School Students.” Telematics and Informatics 57: 101516. 10.1016/j.tele.2020.101516.

[jad12454-bib-0017] Dredge, R. , and L. Schreurs . 2020. “Social Media Use and Offline Interpersonal Outcomes During Youth: A Systematic Literature Review.” Mass Communication and Society 23, no. 6: 885–911. 10.1080/15205436.2020.1810277.

[jad12454-bib-0018] Fabris, M. A. , D. Marengo , C. Longobardi , and M. Settanni . 2020. “Investigating the Links Between Fear of Missing Out, Social Media Addiction, and Emotional Symptoms in Adolescence: The Role of Stress Associated With Neglect and Negative Reactions on Social Media.” Addictive Behaviors 106: 106364. 10.1016/j.addbeh.2020.106364. 32145495

[jad12454-bib-0019] Fabris, M. A. , M. Settanni , C. Longobardi , and D. Marengo . 2024. “Sense of Belonging at School and on Social Media in Adolescence: Associations With Educational Achievement and Psychosocial Maladjustment.” Child Psychiatry & Human Development 55, no. 6: 1620–1633. 10.1007/s10578-023-01516-x. 36920688 PMC11485285

[jad12454-bib-0020] Fitzpatrick, C. , and E. Boers . 2022. “Developmental Associations Between Media Use and Adolescent Prosocial Behavior.” Health Education & Behavior 49, no. 2: 265–271. 10.1177/10901981211035702. 34605695

[jad12454-bib-0021] Griffiths, M. D. 2013. “Social Networking Addiction: Emerging Themes and Issues.” Journal of Addiction Research & Therapy 4, no. 5: 118–119. 10.4172/2155-6105.1000e118.

[jad12454-bib-0023] Hu, L. , and P. M. Bentler . 1999. “Cutoff Criteria for Fit Indexes in Covariance Structure Analysis: Conventional Criteria Versus New Alternatives.” Structural Equation Modeling: A Multidisciplinary Journal 6, no. 1: 1–55. 10.1080/10705519909540118.

[jad12454-bib-0024] Hu, X. , Y. Chen , Z. Wang , E. S. Huebner , and Y. Ling . 2023. “A Latent Transition Analysis of Internet Addiction in Early Adolescents and Its Contributing Factors.” Journal of Early Adolescence 43, no. 5: 603–631. 10.1177/02724316221116045.

[jad12454-bib-0025] Hussain, Z. , K. Kircaburun , M. Savcı , and M. D. Griffiths . 2023. “The Role of Aggression in the Association of Cyberbullying Victimization With Cyberbullying Perpetration and Problematic Social Media Use Among Adolescents.” Journal of Concurrent Disorders. 10.54127/AOJW5819.

[jad12454-bib-0026] Katz, E. , J. G. Blumler , and M. Gurevitch . 1973. “Uses and Gratifications Research.” Public Opinion Quarterly 37, no. 4: 509–523.

[jad12454-bib-0027] Kim, J. , and A. H. N. Cillessen . 2023. “Peer Community and Teacher Closeness as Moderators of the Association Between Peer Status and Aggression.” The Journal of Early Adolescence 43, no. 8: 1043–1070. 10.1177/02724316221142254.

[jad12454-bib-0028] Ko, C. H. , J. Y. Yen , S. C. Liu , C. F. Huang , and C. F. Yen . 2009. “The Associations between Aggressive Behaviors and Internet Addiction and Online Activities in Adolescents.” Journal of Adolescent Health 44, no. 6: 598–605. 10.1016/j.jadohealth.2008.11.011. 19465325

[jad12454-bib-0028a] Ko, C. H. , T. L. Liu , P. W. Wang , et al. 2014. “The Exacerbation of Depression, Hostility, and Social Anxiety in the Course of Internet Addiction Among Adolescents: A Prospective Study.” Comprehensive psychiatry 55, no. 6: 1377–1384. 10.1016/j.comppsych.2014.05.003. 24939704

[jad12454-bib-0030] Kruglanski, A. W. , M. Ellenberg , E. Szumowska , et al. 2023. “Frustration–Aggression Hypothesis Reconsidered: The Role of Significance Quest.” Aggressive Behavior 49, no. 5: 445–468. 10.1002/ab.22092. 37282763

[jad12454-bib-0032] Kırcaburun, K. , C. M. Kokkinos , Z. Demetrovics , O. Király , M. D. Griffiths , and T. S. Çolak . 2019. “Problematic Online Behaviors Among Adolescents and Emerging Adults: Associations Between Cyberbullying Perpetration, Problematic Social Media Use, and Psychosocial Factors.” International Journal of Mental Health and Addiction 17: 891–908. 10.1007/s11469-018-9894-8.

[jad12454-bib-0032a] Lin, S. , C. Longobardi , F. G. M. Gastaldi , and M. A. Fabris . 2024. “Social Media Addiction and Aggressive Behaviors in Early Adolescents: The Mediating Role of Nighttime Social Media Use and Sleep Quality.” Journal of Early Adolescence 44, no. 1: 41–58. 10.1177/02724316231160142.

[jad12454-bib-0033] Little, T. D. , C. C. Henrich , S. M. Jones , and P. H. Hawley . 2003. “Disentangling the “Whys” From the “Whats” of Aggressive.” International Journal of Behavioral Development 27, no. 2: 122–133. 10.1080/01650250244000128.

[jad12454-bib-0034] Longobardi, C. , L. Badenes‐Ribera , M. A. Fabris , A. Martinez , and S. D. McMahon . 2019. “Prevalence of Student Violence Against Teachers: A Meta‐Analysis.” Psychology of Violence 9, no. 6: 596–610. 10.1037/vio0000202.

[jad12454-bib-0035] Longobardi, C. , M. A. Fabris , L. E. Prino , and M. Settanni . 2021. “Online Sexual Victimization Among Middle School Students: Prevalence and Association With Online Risk Behaviors.” International Journal of Developmental Science 15, no. 1–2: 39–46. 10.3233/DEV-200300.

[jad12454-bib-0036] Longobardi, C. , L. E. Prino , M. A. Fabris , and M. Settanni . 2019. “Violence in School: An Investigation of Physical, Psychological, and Sexual Victimization Reported By Italian Adolescents.” Journal of School Violence 18, no. 1: 49–61. 10.1080/15388220.2017.1387128.

[jad12454-bib-0037] Longobardi, C. , M. Settanni , M. A. Fabris , and D. Marengo . 2020. “Follow or Befollowed: Exploring the Links Between Instagram Popularity, Social Media Addiction, Cyber Victimization, and Subjective Happiness in Italian Adolescents.” Children and Youth Services Review 113: 104955. 10.1016/j.childyouth.2020.104955.

[jad12454-bib-0038] LoParo, D. , A. C. Fonseca , A. P. Matos , and W. E. Craighead . 2023. “A Developmental Cascade Analysis of Peer Rejection, Depression, Anxiety, and Externalizing Problems from Childhood Through Young Adulthood.” Research on Child and Adolescent Psychopathology 51, no. 9: 1303–1314. 10.1007/s10802-023-01053-0. 37052808

[jad12454-bib-0039] Low, S. , J. R. Polanin , and D. L. Espelage . 2013. “The Role of Social Networks in Physical and Relational Aggression Among Young Adolescents.” Journal of Youth and Adolescence 42: 1078–1089. 10.1007/s10964-013-9933-5. 23504600

[jad12454-bib-0040] Marengo, D. , M. Angelo Fabris , C. Longobardi , and M. Settanni . 2022. “Smartphone and Social Media Use Contributed to Individual Tendencies Towards Social Media Addiction in Italian Adolescents During the COVID‐19 Pandemic.” Addictive Behaviors 126: 107204. 10.1016/j.addbeh.2021.107204. 34875508

[jad12454-bib-0041] Marengo, D. , M. A. Fabris , L. E. Prino , M. Settanni , and C. Longobardi . 2021. “Student‐Teacher Conflict Moderates the Link Between Students’ Social Status in the Classroom and Involvement in Bullying Behaviors and Exposure to Peer Victimization.” Journal of Adolescence 87: 86–97. 10.1016/j.adolescence.2021.01.005. 33545582

[jad12454-bib-0042] Marengo, D. , M. Settanni , M. A. Fabris , and C. Longobardi . 2021. “Alone, Together: Fear of Missing Out Mediates the Link Between Peer Exclusion in Whatsapp Classmate Groups and Psychological Adjustment in Early‐Adolescent Teens.” Journal of Social and Personal Relationships 38, no. 4: 1371–1379. 10.1177/0265407521991917.

[jad12454-bib-0043] Marsee, M. A. , and P. J. Frick . 2007. “Exploring the Cognitive and Emotional Correlates to Proactive and Reactive Aggression in a Sample of Detained Girls.” Journal of Abnormal Child Psychology 35: 969–981. 10.1007/s10802-007-9147-y. 17636437

[jad12454-bib-0043a] Morese, R. , M. A. Fabris , C. Longobardi , and D. Marengo . 2024. “Involvement in Cyberbullying Events and Empathy are Related to Emotional Responses to Simulated Social Pain Tasks.” Digital health 10: 20552076241253085. 10.1177/20552076241253085. 38766363 PMC11100401

[jad12454-bib-0044] McDonald, R. P. , and M.‐H. R. Ho . 2002. “Principles and Practice in Reporting Structural Equation Analyses.” Psychological Methods 7, no. 1: 64–82. 10.1037/1082-989X.7.1.64. 11928891

[jad12454-bib-0045] Michikyan, M. , and C. Suárez‐Orozco . 2016. “Adolescent Media and Social Media Use: Implications for Development.” Journal of Adolescent Research 31, no. 4: 411–414. 10.1177/0743558416643801.

[jad12454-bib-0046] Monacis, L. , V. De Palo , M. D. Griffiths , and M. Sinatra . 2017. “Social Networking Addiction, Attachment Style, and Validation of the Italian Version of the Bergen Social Media Addiction Scale.” Journal of Behavioral Addictions 6, no. 2: 178–186. 10.1556/2006.6.2017.023. 28494648 PMC5520120

[jad12454-bib-0047] Muthén, L. K. , and B. O. Muthén . 1998–2017. Mplus User's Guide, 8th ed. Los Angeles, CA: Muthén & Muthén.

[jad12454-bib-0048] O'Dell, C. , N. E. Charles , and C. T. Barry . 2024. “Gender Differences in Links between Antisocial Features and Forms and Functions of Aggression Among at‐Risk Youth.” Journal of psychopathology and behavioral assessment 46: 357–372. 10.1007/s10862-024-10134-3.

[jad12454-bib-0050] Patton, D. U. , J. S. Hong , M. Ranney , et al. 2014. “Social Media as a Vector for Youth Violence: A Review of the Literature.” Computers in Human Behavior 35: 548–553. 10.1016/j.chb.2014.02.043.

[jad12454-bib-0051] Pellegrini, A. D. 2002. “Affiliative and Aggressive Dimensions of Dominance and Possible Functions During Early Adolescence.” Aggression and Violent Behavior 7, no. 1: 21–31. 10.1016/s1359-1789(00)00033-1.

[jad12454-bib-0052] Popat, A. , and C. Tarrant . 2023. “Exploring Adolescents’ Perspectives on Social Media and Mental Health and Well‐Being—A Qualitative Literature Review.” Clinical child psychology and psychiatry 28, no. 1: 323–337. 10.1177/13591045221092884. 35670473 PMC9902994

[jad12454-bib-0053] Prinstein, M. J. , J. Boergers , and E. M. Vernberg . 2001. “Overt and Relational Aggression in Adolescents: Social‐Psychological Adjustment of Aggressors and Victims.” Journal of Clinical Child & Adolescent Psychology 30, no. 4: 479–491. 10.1207/S15374424JCCP3004_05. 11708236

[jad12454-bib-0054] Prinstein, M. J. , and A. H. Cillessen . 2003. “Forms and Functions of Adolescent Peer Aggression Associated With High Levels of Peer Status.” Merrill‐Palmer Quarterly 49, no. 3: 310–342. 10.1353/mpq.2003.0015.

[jad12454-bib-0055] Proverbio, A. M. , A. Zani , and R. Adorni . 2008. “Neural Markers of a Greater Female Responsiveness to Social Stimuli.” BMC Neuroscience 9: 56. 10.1186/1471-2202-9-56. 18590546 PMC2453130

[jad12454-bib-0056] Radovic, A. , T. Gmelin , B. D. Stein , and E. Miller . 2017. “Depressed Adolescents’ Positive and Negative Use of Social Media.” Journal of Adolescence 55: 5–15. 10.1016/j.adolescence.2016.12.002. 27997851 PMC5485251

[jad12454-bib-0057] Rustamov, E. , M. Aliyeva , N. Rustamova , U. Z. Nuriyeva , and U. Nahmatova . 2023. “Aggression Mediates Relationships between Social Media Addiction and Adolescents’ Wellbeing.” Open Psychology Journal 16, no. 3: 1–7. 10.2174/0118743501251575230925074655.

[jad12454-bib-0058] Savcı, M. , and F. Aysan . 2018. “# Interpersonal Competence, Loneliness, Fear of Negative Evaluation, and Reward and Punishment as Predictors of Social Media Addiction and Their Accuracy in Classifying Adolescent Social Media Users and Non‐Users.” Addicta: The Turkish Journal on Addictions 5, no. 3: 431–471. 10.15805/addicta.2018.5.3.0032.

[jad12454-bib-0059] Selig, J. P. , and T. D. Little . 2012. “Autoregressive and Cross‐Lagged Panel Analysis for Longitudinal Data.” In Handbook of Developmental Research Methods, edited by B. Laursen , T. D. Little , and N. A. Card , 265–278. New York: The Guilford Press.

[jad12454-bib-0060] Shannon, H. , K. Bush , P. J. Villeneuve , K. G. Hellemans , and S. Guimond . 2022. “Problematic Social Media Use in Adolescents and Young Adults: Systematic Review and Meta‐Analysis.” JMIR Mental Health 9, no. 4: e33450. 10.2196/33450. 35436240 PMC9052033

[jad12454-bib-0061] Smith, R. L. , A. J. Rose , and R. A. Schwartz‐Mette . 2010. “Relational and Overt Aggression in Childhood and Adolescence: Clarifying Mean‐Level Gender Differences and Associations With Peer Acceptance.” Social Development 19, no. 2: 243–269. 10.1111/j.1467-9507.2009.00541.x. PMC2855554 20401342

[jad12454-bib-0062] Su, W. , X. Han , H. Yu , Y. Wu , and M. N. Potenza . 2020. “Do Men Become Addicted to Internet Gaming and Women to Social Media? A Meta‐Analysis Examining Gender‐Related Differences in Specific Internet Addiction.” Computers in Human Behavior 113: 106480. 10.1016/j.chb.2020.106480.

[jad12454-bib-0064] Vannucci, A. , E. G. Simpson , S. Gagnon , and C. M. Ohannessian . 2020. “Social Media Use and Risky Behaviors in Adolescents: A Meta‐Analysis.” Journal of Adolescence 79: 258–274. 10.1016/j.adolescence.2020.01.014. 32018149

[jad12454-bib-0066] Wang, C. , X. Li , and L. X. Xia . 2023. “Long‐Term Effect of Cybervictimization on Displaced Aggressive Behavior Across Two Years: Mutually Predicting Mediators of Hostile Emotion and Moral Disengagement.” Computers in Human Behavior 141: 107611. 10.1016/j.chb.2022.107611.

[jad12454-bib-0067] Wong, N. , T. Yanagida , C. Spiel , and D. Graf . 2022. “The Association Between Appetitive Aggression and Social Media Addiction Mediated By Cyberbullying: The Moderating Role of Inclusive Norms.” International Journal of Environmental Research and Public Health 19, no. 16: 9956. 10.3390/ijerph19169956. 36011592 PMC9407729

[jad12454-bib-0068] Xing, J. , M. Peng , Z. Deng , K. L. Chan , Q. Chang , and R. Ho . 2023. “The Prevalence of Bullying Victimization and Perpetration Among the School‐Aged Population in Chinese Communities: A Systematic Review and Meta‐Analysis.” Trauma, Violence & Abuse 24, no. 5: 3445–3460. 10.1177/15248380221129595. 36331136

[jad12454-bib-0069] Xu, X. P. , Q. Q. Liu , Z. H. Li , and W. X. Yang . 2022. “The Mediating Role of Loneliness and the Moderating Role of Gender Between Peer Phubbing and Adolescent Mobile Social Media Addiction.” International Journal of Environmental Research and Public Health 19, no. 16: 10176. 10.3390/ijerph191610176. 36011810 PMC9407745

[jad12454-bib-0070] Yue, X. , and Q. Zhang . 2023. “The Association Between Peer Rejection and Aggression Types: A Meta‐Analysis.” Child Abuse & Neglect 135: 105974. 10.1016/j.chiabu.2022.105974. 36521401

